# Views and experiences of community pharmacy team members on antimicrobial stewardship activities in Scotland: a qualitative study

**DOI:** 10.1007/s11096-020-01042-z

**Published:** 2020-08-16

**Authors:** Antonella Pia Tonna, Anita Elaine Weidmann, Jacqueline Sneddon, Derek Stewart

**Affiliations:** 1 School of Pharmacy and Life Sciences, Robert Gordon University, Aberdeen, AB10 7AQ Scotland, UK; 2 Scottish Antimicrobial Prescribing Group, Healthcare Improvement Scotland, Delta House, 50 West Nile Street, Glasgow, G1 2NP Scotland, UK; 3 College of Pharmacy, Qatar University Health, Qatar University, PO Box 2713, Doha, Qatar

**Keywords:** Antimicrobial stewardship, Community pharmacy, European antibiotic awareness day, Qualitative methods, Scotland

## Abstract

*Background* It has been acknowledged and recognised internationally that the community pharmacy team has a major role to play in antimicrobial stewardship programmes, particularly regarding patient engagement. However, there is a paucity of published research on community pharmacy-based activities in antimicrobial stewardship, and views and perceptions of the community pharmacy team on their role in antimicrobial stewardship. *Objective* To explore views and experiences of community pharmacy teams across Scotland on antimicrobial stewardship, activities related to European Antibiotic Awareness Day, and a self-help guide to treating infection. *Setting* Community pharmacy, Scotland. *Methods* Qualitative, semi-structured in-depth telephone interviews were undertaken with a purposive sample of community pharmacy team members over a six week period between November and December in 2016. Interviews were audio-recorded, transcribed verbatim and data analysed thematically using the framework approach. *Main outcome measure* Views and perceptions of antimicrobial stewardship and European Antibiotic Awareness Day activities and role of the pharmacy team. *Results* Twenty-seven participants were interviewed—20 pharmacists, five pharmacy graduates completing their pre-registration year, and members of the pharmacy support team including two pharmacy technicians and one medicines counter assistant. They were working mainly in urban areas and across five regions of Scotland. Most were aware of antimicrobial stewardship but some were not familiar with the term. Participants identified roles for the community pharmacy team in antimicrobial stewardship including the importance of the pharmacy as a first port of call for self-care advice. Some participants, including pharmacists, showed lack of awareness of European Antibiotic Awareness Day; those who were aware thought it may not have the desired impact on educating the public. Most participants, irrespective of role within the team, were not familiar with the self-help guide but they perceived this as a useful resource for the pharmacy team. *Conclusion* The participants recognised and identified roles for the community pharmacist within antimicrobial stewardship. However, the lack of awareness of European Antibiotic Awareness Day shows a need for European Antibiotic Awareness Day tools and other materials to be more effectively disseminated and for more training to be provided.

## Impacts on practice

- The research indicates a need for further education of the community pharmacy team to better engage with the public about self-limiting infections
- The lack of awareness of the self-help guide indicates a need for broader dissemination of and education about this resource within the community pharmacy team

## Introduction

Antimicrobial resistance (AMR) has been identified as a global threat with resistant related infections causing at least 50,000 premature deaths in the United States (US) and Europe [1]. AMR develops when microorganisms become resistant to antimicrobials and may be due to factors including the inappropriate and unnecessary use of antimicrobials [2]. The World Health Organization (WHO) Global Action Plan describes antimicrobial stewardship (AMS) initiatives to retard resistance development [2]. World Antibiotic Awareness Week (WAAW), launched by WHO in 2015, is a global campaign which aims “to increase awareness of global antibiotic resistance and to encourage best practices among the general public, health workers and policy makers to avoid the further emergence and spread of antibiotic resistance [3].”

European Antibiotic Awareness Day (EAAD), a European wide public-professional partnership that commenced in 2008, has now been integrated within WAAW [4]. In the United Kingdom (UK), EAAD is led by Public Health England and supported by Scotland, Wales and Northern Ireland and many professional organisations. Campaigns have focused mainly on reducing unnecessary antibiotic use for self-limiting respiratory infections utilising posters and leaflets to support engagement of health and care staff across all settings, patients and the public.

There is evidence that standalone resources are unlikely to be effective in changing public behaviours and should be combined with approaches such as targeted campaigns and reinforcement [5]. Given that community pharmacies are often the first point of contact for treatment of self-limiting conditions, these could be an ideal setting for targeted campaigns and professional reinforcement. This role has been supported by international organisations including the International Pharmaceutical Federation and the Pharmaceutical Group of the European Union [6, 7]. Internationally, the potential contribution of community pharmacists to reducing AMR has been recognised with emphasis on the community pharmacist as a key public health educator on AMR and infection control [8]. This is especially so when raising public awareness that antibiotics are usually not required for common respiratory infections [6, 7]. Best practice examples of health promotion events and campaigns by community pharmacists are described in the international literature [6, 7]. However, a systematic review of community pharmacists’ contributions to public health did not identify any research relating community pharmacists to AMS and AMR activities [9]. Nothwitstanding this potential for the community pharmacist contribution, knowledge of and perceptions of community pharmacists about this area of practice is very limited [10].

**Fig. 1:**
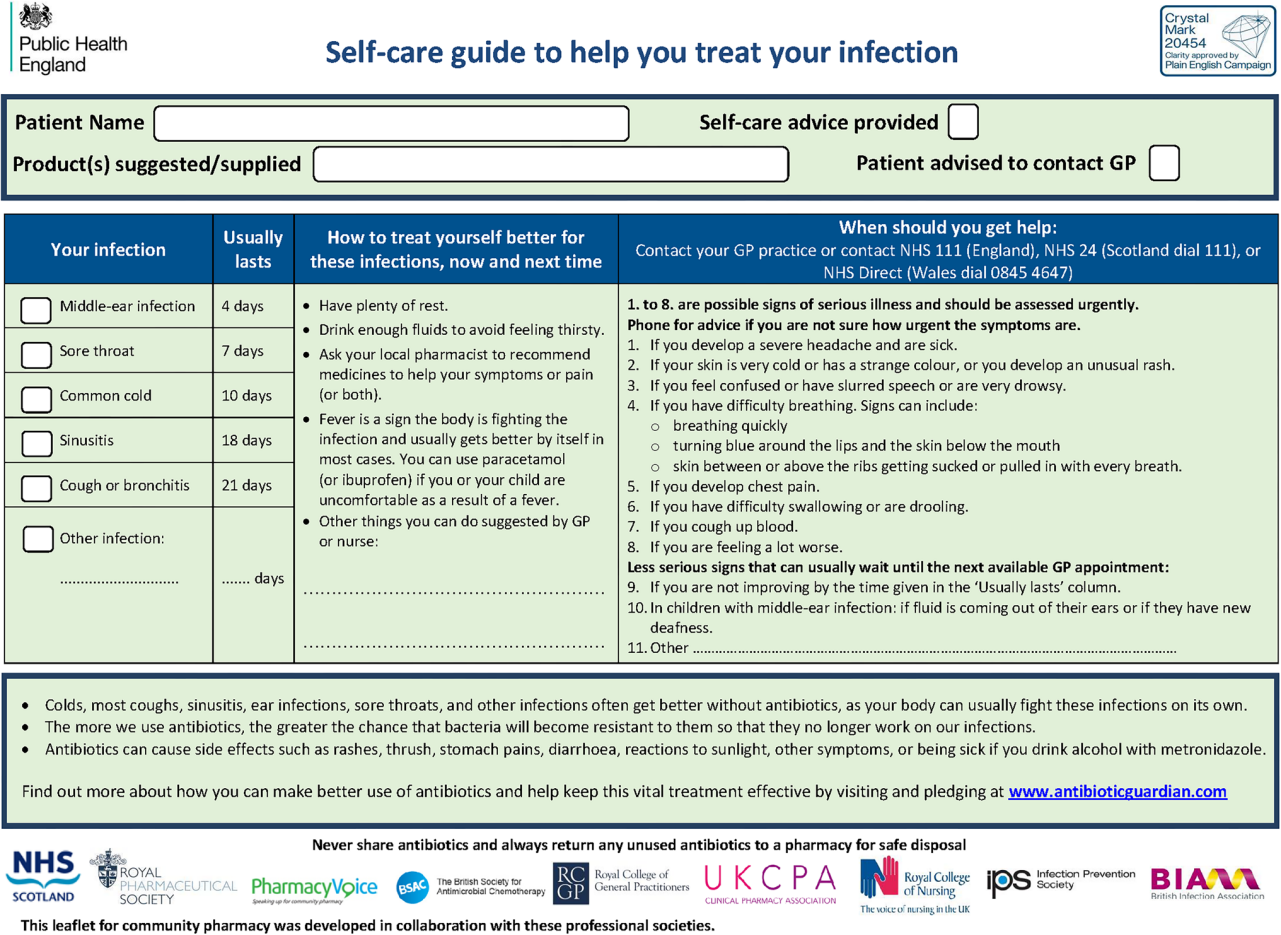
Community pharmacy version of “Self help guide to treating your infection [9]”

Community pharmacy teams across Scotland have been actively encouraged and supported by the Scottish Antimicrobial Prescribing Group (SAPG) to contribute to EAAD. SAPG is a multidisciplinary group set up in 2008 and aimed at co-ordinating efforts on AMS in Scotland. Since 2014 this has included an initiative to utilise a community pharmacy version of a “Self-help guide to treating your infection” leaflet developed by the Royal College of General Practitioners (RCGP) [11] (Fig. 1). In 2014, printed copies of this resource together with a covering letter were distributed to all community pharmacies by SAPG, to coincide with EAAD and a training evening on AMS hosted by NHS Education for Scotland, was delivered in all regions. The community pharmacy version of the leaflet is now available via the RCGP website and in Scotland community pharmacies can request printed supplies of the leaflet to use throughout the year [11]. There has been limited evaluation of the self-help guide or indeed of EAAD materials in a community pharmacy setting and this research aimed to fill the gap.

## Aim of the study

To explore the views and experiences of community pharmacy teams across Scotland on AMS, on activities related to EAAD and on use of the “Self-help guide to treating your infection” leaflet.

## Ethics approval

Approval was obtained from the Robert Gordon University, School of Pharmacy and Life Sciences, Ethics Committee. As a service evaluation, this study was exempt from NHS Ethics and Management Review.

## Methods

The COREQ checklist aimed at ensuring the quality of reporting of qualitative research, was used to guide the reporting of this research [12].

### Design

This was a qualitative study comprising telephone-based semi-structured interviews with pharmacy team members throughout Scotland. A research team of pharmacists with expertise in AMS, community pharmacy practice and qualitative research was established to oversee the study. The interviews were conducted by final year MPharm undergraduate students who were trained and provided with supervision and support. Telephone rather than face-to-face interviews were selected due to logistic and resource reasons, allowing recruitment throughout Scotland and permitting interviews to be conducted outside working hours.

### Recruitment

Since all community pharmacy team members, irrespective of their role are central to AMS and likely to interact with patients on aspects of AMS, all were invited to participate. The community pharmacy team comprises pharmacists, pharmacy graduates undertaking a one year internship, pharmacy technicians (members of team who dispense medication and provide advice to patients), and medicines counter assistants (members of team who provide advice about over-the-counter medication). A letter inviting participation and a participant information leaflet were sent via email by Community Pharmacy Scotland (an organisation that represents community pharmacy owners and their teams throughout Scotland) to all community pharmacies in Scotland to be distributed to all staff members [13]. Two reminders were sent out at two weekly intervals. Those interested in participating were requested to return electronically to the research team a completed consent form and short questionnaire collecting data on demographics and pharmacy role.

### Development of data generation tool

A semi-structured interview schedule was developed by the authors some of whom are AMS experts, following a literature review on information within EAAD resources [4]. This was reviewed for credibility by the research team. The schedule was piloted in face-to-face interviews with three community pharmacy team members. As no changes were made to the interview schedule post-pilot, the data generated from the pilot interviews were included in the final dataset. Table 1 gives the outline of the interview schedule.

**Table 1:** Semi-structured interview schedule

Key items	Prompts to elicit more in-depth response
*Topic 1: Antimicrobial stewardship*	
Are you aware of the term “antimicrobial stewardship” and why it is important?	Can you elaborate further? Why do you think antimicrobial stewardship is important? Have you undergone any specific training relating to antimicrobial stewardship?
*Topic 2: Role of community pharmacy team in antimicrobial stewardship*	
In your opinion, is there a role for the community pharmacist and their team to contribute towards antimicrobial stewardship?	If no, what can be done so that you as a community pharmacist could play a larger role in antimicrobial stewardship? Can you give examples of ways that the community pharmacist and their team can contribute towards antimicrobial stewardship? Are there any specific examples that you can provide us with from your practice?
*Topic 3: Views and perceptions of EAAD*	
Are you familiar with European Antibiotic Awareness Day (EAAD)?	What do you know about this day? Why has this day developed?
Do you recall your pharmacist being sent a package containing EAAD resources for use?	Did your pharmacy keep these activities and resources? Are these activities kept someplace accessible in your pharmacy? Are you aware of what was included in the package? Did seeing the resources/activities highlight any additional learning needs for yourself?
Are you familiar with any other resources used to promote prudent use of antibiotics as part of the EAAD, such as promotional leaflets and posters or educational quizzes?	If not, why not? What are the leaflet’s limitations? Did these limitations prevent it from being distributed in your pharmacy? Which pharmacy team member would you say distributed the majority of leaflets? E.g. pharmacist Which was the most common patient group and age range receiving most leaflets? Approximately how many?
*Topic 4: Views and perceptions of self-help guide leaflet*	
Do you recall if the self-help guide leaflet was distributed to any patients or caregivers in your pharmacy?	If not, why not? Do you have any recommendations for improving this section of the leaflet?
Overall, did you find the self-help guide leaflet a useful tool within the pharmacy?	Can you recall anything in particular that is confusing for patients or caregivers? Were patients able to understand the content? Which patient age range would you say best understands the scope of the leaflet?
Was the information provided in the self-help guide leaflet different in any way to information that would be usually provided to patients or caregivers in the pharmacy?	Are these appropriate at promoting proper antibiotic usage by patients? Which in particular are most useful/liked by patients? Do you have any comments you would like to share with us about these additional resources?
Did you find the patient specific advice at the top of the self-help leaflet useful?	
In your opinion, is the self-help guide clear and understandable to the general public?	
Did you receive any feedback, positive or negative from patients and carers about the self-help guide leaflet?	
Did you receive any feedback, positive or negative, from other members of your pharmacy team about the self-help guide leaflet?	
Would you be keen to use the self-help leaflet on an ongoing basis?	
Do you have any other comments about the self-help leaflet you would like to share with us?	

### Data generation

All those returning the consent form (n = 28) were included in the study. Interviews were conducted over a six-week period between November and December 2016, recorded digitally, and transcribed verbatim, with a member of the research team checking all transcripts for transcribing accuracy.

### Data analysis

Transcripts from all interviews were independently analysed for content by the students who conducted the interviews and again independently by two researcher members of the team using the Framework Approach of: data familiarisation; identifying constructs, indexing; charting, mapping and interpreting [14].

## Results

### Demographics

Of the 28 interviews, only 27 transcripts were available for analysis due to one recording failure. Interviews lasted between 15 and 30 min. The 27 were largely female (n = 22), represented five health board regions in Scotland and practised in urban areas (n = 26). Twenty interviewees were pharmacists (two were independent prescribers), five were pharmacy graduates completing their pre-registration internship year and three were non-pharmacist team members (2 pharmacy technicians, 1 medicines counter assistant).

Four main themes were identified from the interviews are summarised in Table 2.

**Table 2:** Themes identified from the interviews

Interview schedule topic	Themes
Antimicrobial stewardship	Appropriate use of antimicrobials
	Link with antimicrobial resistance
	AMS initiatives
Role of community pharmacy team in antimicrobial stewardship	Provision of reassurance and advice
	Pharmacy team AMS post-graduation training
Views and perceptions of EAAD	Lack of awareness of EAAD
	Perceived effectiveness of pharmacy in promoting EAAD
Views and perceptions of “Self help guide to treating your infection”	Lack of familiarity with self help guide
	Perceived usefulness of self help guide
	Recommendations for future iterations of self help guide

Themes are presented aligned to the topics of the interview schedule.

### Topic 1: antimicrobial stewardship

#### Appropriate use of antimicrobials

Most interviewees linked AMS with ensuring appropriate use of antimicrobials and these being used only when indicated.

It’s really just being in control of antibiotics and only giving them out where and when they are deemed most necessary. (P16, pharmacist).

However, a few, irrespective of the role within the community pharmacy team, were not familiar with the term antimicrobial stewardship,

No I’ve never heard of that (P27, pharmacist) and I have not really no (P21, pharmacy graduate).

To facilitate completion of the interviews, participants were provided with a prompt defining antimicrobial stewardship.

#### Link with antimicrobial resistance

Interviewees also recognised AMS as being necessary to reduce resistance to antimicrobials.

Using antibiotics appropriately i.e. using them when they are needed and not using them when they are not needed to prevent antimicrobial resistance. (P19, pharmacist).

Two interviewees, both of whom were pharmacists, had misconceptions on antimicrobial resistance development inaccurately stating that the patients developed resistance rather than the microorganisms, for example.

I would think it [antimicrobial stewardship] would mean making sure that people are given antibiotics appropriately and not getting them when they don’t really need them**, so that people don’t build up resistance** [incorrect statement in bold]. (P1, pharmacist).

#### Antimicrobial stewardship initiatives

Interviewees considered AMS as a collective responsibility that should be dealt with on a global scale by the wider care team.

… it’s the responsibility of all health care professionals to ensure that antibiotics are used in a responsible way. (P4, pharmacist).

… antimicrobial stewardship is a global initiative … ultimately it affects all of us. (P3, pharmacist, independent prescriber).

### Topic 2: role of community pharmacy team in antimicrobial stewardship

#### Provision of reassurance and advice

Interviewees highlighted a proactive role for the community pharmacy team in setting patient expectations around no need for antibiotics to treat self-limiting conditions, and greater emphasis on self-care.

I think that’s one of the areas that we can excel in … as pharmacists, it’s our role to act as stewards for antibiotics, particularly in Scotland where we are the first call for a lot of people with minor ailments so what we can do is give appropriate self-care advice and reassurance to the patients that antibiotics aren’t required … (P28, pharmacist).

Specific examples of self-limiting conditions included viral infections with symptoms of coughs and colds, minor skin infections, ear infections, conjunctivitis and urinary tract infections.

You get people coming in everyday … they might have the start of what they think might be a UTI … we can give them advice like drink plenty of water, try flush out or try cranberry cystitis sachets before going and getting antibiotics. (P16, pharmacist).

#### Pharmacy team AMS post-graduation training

Most interviewees could not recall having received specific training on AMS post-completion of their undergraduate degree.

I have not really, no. We were kind of made aware of it at one of the NES [NHS Education for Scotland] training days but I wouldn’t say it was training on stewardship or anything, no. (P21, pharmacist graduate).

In contrast, the two pharmacist independent prescribers indicated that regular training relating to their prescribing role was provided.

… throughout the four years that I did unscheduled care, [prescribing role] there was a continual thing [training]. (P3, pharmacist, independent prescriber).

Some pharmacists voiced the importance of training and supporting other members of the pharmacy team. They were aware that counter staff were most likely to be the first point of contact in the pharmacy.

You also have to watch your staff … it’s not just us being educated; it’s about the staff that speak to customers before you get there. (P10, pharmacist).

### Topic 3: views and perceptions of EAAD

#### Lack of awareness of European Antibiotic Awareness Day

Though most interviewees were aware of EAAD, they were unable to describe the aim and content of the resources in detail.

Aware in the vaguest of ways … so I wouldn’t know when it was but I know there was one. (P19, pharmacist).

Some interviewees, including all non-pharmacist team members, had never heard of EAAD and answered “No,” (P23, pharmacy technician) “No, not really.” (P26, medicines counter assistant).

#### Perceived effectiveness of pharmacy in promoting EAAD

Several perceived the community pharmacy as being effective in promoting EAAD by providing the public with information.

…was very well sign posted and we had a big poster in the window and we had leaflets out (P3, pharmacist, independent prescriber).

Others, however, noted that there was potential for information overload with loss of impact.

… they [posters] kind of get lost with all the other posters … it’s another poster … you could put up hundreds, it’s not easy. (P25, pharmacist).

Several also noted that the resources were unlikely to have impact particularly for those with a perceived need for an antibiotic.

… if someone doesn’t feel well and they just want to feel better they’re going to want an antibiotic. So whether you put a poster up or not, I don’t think it’s going to make any difference. (P27, pharmacist).

### Topic 4: views and perceptions of “Self help guide to treating your infection” leaflet

#### Lack of familiarity with self help guide

Overall, there was a general lack of familiarity with the guide. Some interviewees were aware of the guide, prior to being sent one as part of this research recruitment pack. Not all had seen the guide in their community pharmacy; some were familiar via other sources such as attending an AMS training session.

I have seen something like that [self-help guide]. It could have been at that antibiotic stewardship event but I don’t remember it coming directly to the pharmacy. (P4, pharmacist).

#### Perceived usefulness of self-help guide

Most participants did not use the guide within the pharmacy. A few had integrated information from the self-help guide within their practice when providing advice to patients.

It certainly helped to explain it to patients and when they have it written down it’s almost like they believe you slightly more, than if you are just telling them. (P25, pharmacist).

[handed out to] People who I feel particularly wanted antibiotics and I didn’t think it [the antibiotic] was appropriate. (P27, pharmacist).

Participants who viewed the self-help guide while participating in this interview were overwhelmingly positive indicating that the information provided allowed pharmacy team members to consistently and systematically provide advice to patients. Participants believed that the guide could be effectively integrated with the verbal advice provided and acting as a useful resource for patients to take home to re-inforce key messages.

… it’s quite a good reminder of the points to bring up rather than just sort of winging and going with what’s in your own head. (P9, pharmacist).

… it helps for staff training, to raise awareness amongst them … to make sure they are giving a consistent message and it’s not just me that’s saying “You don’t need an antibiotic.” (P13, pharmacist).

Participants also thought the leaflet may support pharmacy staff in communicating more effectively with patients if their condition does not require an antibiotic.

If somebody says they’ve got the cold or flu and are wanting to know how roughly long it would last it is nicer having something in black and white to say “Look this is how long it could potentially last … “ (P16, pharmacist).

#### Recommendations for future iterations of self-help guide

Many participants thought there was too much information in the guide, considered the format to be very busy and the font too small for some patients to read.

It’s a bit busy, it is not written in a format that would just be advice … It looks a bit too much like something official, like an actual form. I think it doesn’t engage the reader quite as well as it could. (P13, pharmacist).

Participants made suggestions for improvement including changes to the layout, providing various formats including bigger fonts and translations into other languages, and availability of the leaflet electronically. Other recommendations included providing out-of-hours service contacts and clearer guidance on action to take if patient hypothermic.

If it was made in different formats because it’s only printed in English … or we might have visually impaired patients so if it’s kind of in a bigger format or electronic so we could email or send out to people; that would be useful. (P20, pharmacist).

Participants also proposed more effective dissemination, with an all year round provision rather than this being linked only to EAAD.

I think at the time when we looked at the leaflet or the non-prescription pad we thought it was a really good idea; but then actually we didn’t get any copies of it or have access to if afterwards [EAAD]. (P4, pharmacist).

## Discussion

Key findings of this study showed that many of the participants in this study were aware of AMS, how this was linked to appropriate use of antimicrobials and the implications that this has in reducing antimicrobial resistance. However, some were not familiar with the term and a few had misconceptions about development of resistance. Various roles were identified for the community pharmacy team to contribute to AMS with emerging themes including the importance of the pharmacy as a first port of call with the pharmacy team ideally placed to provide patients with reassurance and advice. It was disappointing to note some participants’ lack of awareness of EAAD and that those who knew about it suggested it may not have the desired impact on educating the public. Despite the fact that most participants were not familiar with the “Self help guide for treating your infection”, they perceived it as a very useful resource to providing patients with information and reinforce the pharmacy team advice.

There are many strengths to this study including the fact that a range of pharmacy team members were interviewed. The qualitative approach allowed for in-depth exploration of participant views and opinions and measures were taken to promote research trustworthiness. Investigator triangulation was implemented with two researchers independently analysing all interviews, and a record and description of the research steps from start to report of findings were kept and are provided to ensure confirmability [15, 16].

There are however a number of limitations to the study. Participants were from only five of the 14 regional health boards and were mainly pharmacists. Therefore, the views and opinions expressed may not be those of all pharmacy team members and any difference between opinions could not be identified. Efforts were made to ensure credibility and responses were confidential thus making every effort not to impact on workplace relationships [16]. The interviews have been carried out more than three years ago; yet there is no evidence to indicate any changes in practice and the self-help guide has not been reviewed. Despite these limitations, this qualitative research has added to the very limited evidence base currently available on community pharmacy engagement with AMS and EAAD.

Provision of self-care advice has been viewed by participants as a major role for the community pharmacy team. This is in line with the ambitions of ‘Achieving excellence in pharmaceutical care: a strategy for Scotland’ and the services contracted via Community Pharmacy Scotland [13, 17]. Both the longstanding Minor Ailments Service and the more recent Pharmacy First service which targets common infections, support and encourage the public to use pharmacies as the ‘first port of call [18].’ In relation to AMS, the National Institute for Health and Care Excellence (NICE) guideline and the *CDC guidance on Core Elements of Outpatient Stewardship*, recommend that community pharmacies are a key partner. This includes setting patient expectations particularly if they are unlikely to need antibiotic treatment [19, 20]. The importance of this is emphasized by evidence demonstrating that perceived patient expectation and pressure are a recognised barrier to AMS in primary care. This is supported by a recent literature review concluding that patients are more likely to be prescribed antibiotics if they request them and are more dissatisfied with their care if they do not receive them [21]. The importance of community pharmacies in the provision of advice on antibiotics is reinforced by the fact that 51% of the UK public consult their pharmacist for advice on antibiotics [22]. Thus it is important that community pharmacies provide robust, standardised messages about self-care and use of antibiotics. All community pharmacy team members require to have knowledge of AMS and confident communication skills. To ensure a consistent approach, all staff members must give consistent messages about antibiotics and the results of this study suggest further training may be required. The self-help guide is a potential tool to ensure such consistent advice and a resource to reinforce advice provided by pharmacists on management of self-limiting infections. The fact that the self-help guide was originally developed by a GP professional body ensures that pharmacists and GPs are providing patients with similar self-care advice [11]. However, this study shows that further work is required to increase uptake and use of the community pharmacy self-help guide.

Training of healthcare professionals in AMS is important to support both the pharmacists’ role but also a team approach and consistent messaging. The importance of providing patient-centred care in relation to AMS was identified as an essential competency for training of undergraduates and UK healthcare professionals new in their role, including pharmacists [23]. Having the confidence to query clinical prescriptions was acknowledged as a potential challenge and area for development for community pharmacists to support implementation of AMS programmes [23]. These findings are also similar to a survey of community pharmacists in England where participants identified a number of barriers to clinically checking antibiotic prescriptions. These included, lack of confidence, a concern that the relationship with the prescribers may be harmed and a lack of knowledge of diagnosis for which antibiotic has been prescribed [24]. The evidence therefore suggests that further training is required and this has been recognized by various groups who have producing further training for community pharmacists [25].

Pharmacies are ideally placed to show commitment to AMS through display of materials such as posters and leaflets providing information about AMS. However, participants in this study did not perceive this approach to be particularly effective.

## Conclusion

This research adds to the small body of research investigating community pharmacists and AMS. It suggests a lack of comprehensive knowledge on AMS among the community pharmacy team members, highlighting a need for further education of these staff to improve engagement with patients around management of self-limiting infections. Further research is required to explore ways in which use of the self-help guide can be more effectively supported to ensure all pharmacy staff have access to and know how to utilise this valuable resource. Furthermore, research is required to explore barriers and facilitators of the community pharmacy team engagement with EAAD and other AMS activities.
